# Pilot investigation into osteotome hard surface coating and cutting-edge degradation

**DOI:** 10.1186/s13018-020-01768-6

**Published:** 2020-07-10

**Authors:** David E. White, Jim Bartley, Christopher Whittington, Lorenzo M. G. Garcia, Kaushik Chand, Celine Turangi

**Affiliations:** 1 BioDesign Lab, School of Engineering, Computer and Mathematical Sciences, Auckland University of Technology, Private Bag 92006, Auckland, 1142 New Zealand; 2 Department of Surgery, University of Auckland, Auckland, New Zealand

**Keywords:** Blade sharpness, Hard coating, Osteotomy, Instrument maintenance, Surgery

## Abstract

**Background:**

Osteotomes are bone cutting tools commonly reused in orthopedic surgical procedures. Despite undergoing rigorous cleaning, visual inspection, and sterilization procedures between every use, the condition of the cutting blade edge is commonly not qualitatively assessed. Subjective feedback from surgeons suggests a large variation in osteotome cutting-edge sharpness is found during use. This study seeks to investigate the retention of osteotome cutting-edge sharpness by comparing the wear resistance of as-supplied, electroless nickel, and titanium nitride coated osteotomes following a series of bone cutting tests.

**Methods:**

Changes in edge sharpness were assessed using visual inspection, depth penetration testing that quantified change in the blade sharpness index, and scanning electron microscopy visual analysis. Visual inspection of each osteotome blade edge was then compared to qualitative blade sharpness index measurement.

**Results:**

After use, no cutting-edge damage or change in blade sharpness was detected by visual examination of all three osteotomes; however, the as-supplied osteotome demonstrated 50% loss of blade sharpness index compared to 30% and 15% reduction for the electroless nickel and titanium nitride coated osteotomes, respectively. This finding was supported by scanning electron microscopy evaluation that found greater mechanical damage had occurred along the cutting edge of the as-supplied osteotome compared to the two coated with wear resistant materials.

**Conclusions:**

The rapid loss of blade sharpness found in the as-supplied osteotome supports the degradation in cutting performance frequently reported by surgeons. The findings from this study demonstrate blade sharpness index better detects cutting-edge wear compared to visual inspection. Results from this pilot study also suggest the coating of osteotomes in hard-wearing biocompatible materials assists in retaining cutting-edge sharpness over multiple uses. Further study using a larger sample size is required to validate these findings.

## Background

Osteotomes are used during surgery where bone needs to be refractured or removed using a chiseling motion. Stainless steel is commonly used in the manufacture of osteotomes, mainly for its low cost, biocompatibility, and ability to resist corrosion. Although this type of steel has multiple positive benefits, it lacks the ability to maintain a sharp edge when used as a cutting tool, compared to other harder metal alloys. Hard surface coatings, such as electroless nickel and titanium nitride (TiN), potentially offer a low-cost solution to this problem.

It is important that the osteotome cutting edge remains sharp throughout the operation to minimize the percussion force required to cut bone. A blunt osteotome could produce bone fragments and bone micro-fractures rather than clean cuts, potentially causing damage to the surrounding soft tissue and leading to poor surgical outcomes [1].

Despite the osteotome commonly receiving visual inspection during post-operative cleaning and sterilization procedures, it is questionable as to how effectively this method assesses cutting-edge wear and damage. For the surgeon, there are a few signs that the osteotome cutting edge is degrading during use. The most apparent indicators of a blunt osteotome are loss of cut accuracy as the tool takes the path of least resistance and more frequent mallet use [2].

This work had two objectives:

1. To determine if the state of osteotome blade sharpness can be accurately assessed by visual inspection.
2. To determine if hard surface coating provides better retention of cutting-edge sharpness compared to an osteotome in the as-supplied state.

## Methods

This study utilized three identical and new commercially available osteotomes (RU 5331-30, Rudolf Medical GmbH, Fridingen, Germany). One osteotome was electroless nickel coated to a thickness of 8 μm, another was TiN coated using physical vapor deposition (PVD) to a thickness of 2 μm, and the remaining one was left in the as-supplied state. Electroless nickel and TiN coatings were each applied at the minimum viable thickness offered by their respective coating processes.

All osteotomes initially underwent visual cutting-edge inspection and blade sharpness index (BSI) testing (described later in more detail), to benchmark cutting-edge sharpness. Each osteotome then underwent a series of bone cutting cycles with the visual and BSI assessments repeated after each of the four bone cutting tests. Upon completion of testing, a small sample of the cutting edge was removed from each osteotome to be subjectively compared for wear and damage using scanning electron microscopy (SEM) techniques (described later).

### Bone cutting protocol

Bone cutting entailed holding the osteotome at an inclination with the aid of a cutting jig that replicated the procedure commonly used during surgery.

**Fig. 1:**
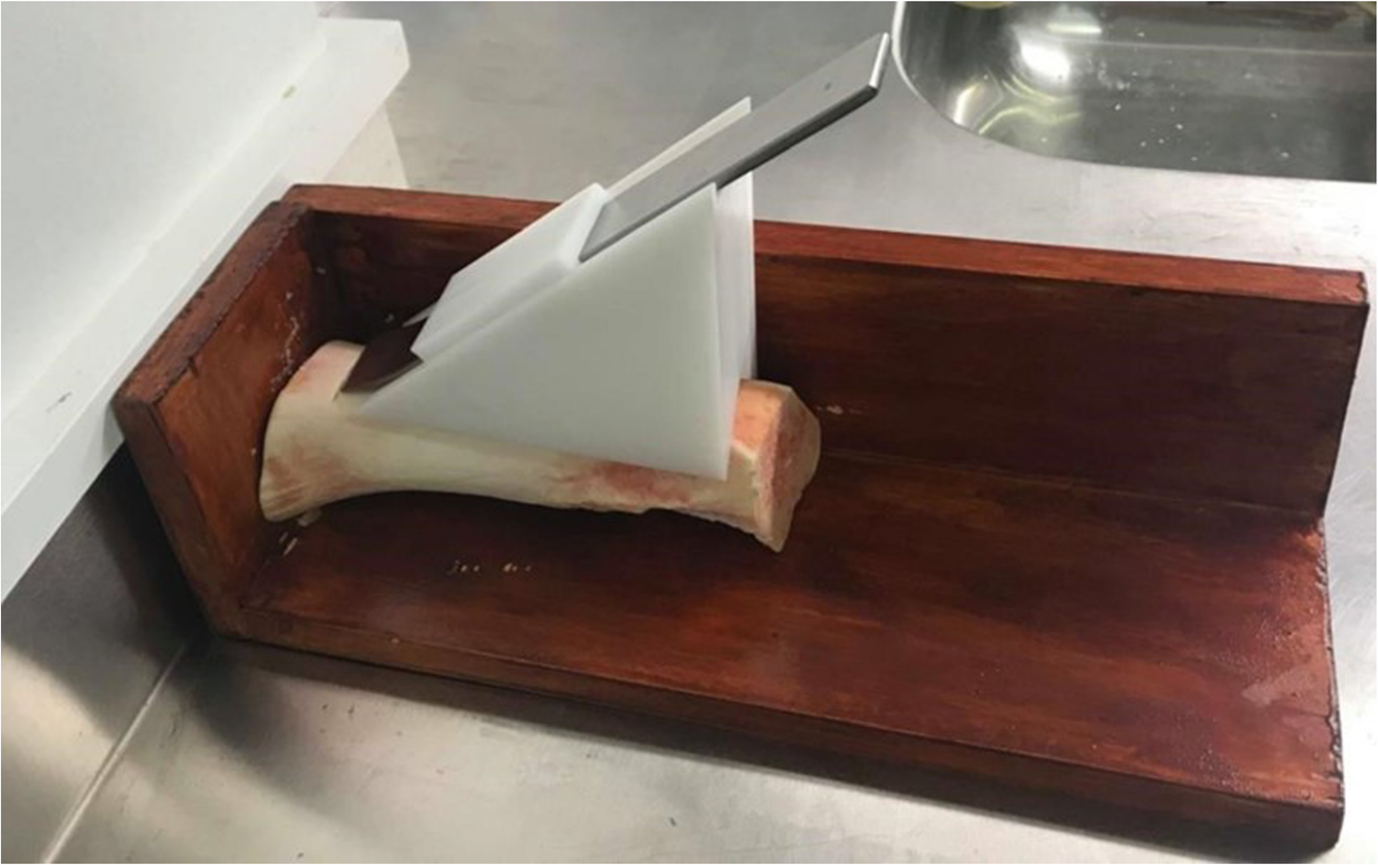
Cutting jig holding osteotome at a controlled angle and cut depth during longitudinal axial cut. Same sectioned controlled length of adult bovine femur bone used for all tests

Each bone cutting cycle removed a 40-mm length of cuticle bone to a depth of 5 mm along the axial direction of the freshly excised adult bovine femur. The same bone sample was utilized to ensure consistency of bone strength between each test. The jig held the bone in position and aligned the osteotome cutting direction along the femur bone longitudinal axis via a lower vee-groove shown in Fig. 1. The cutting jig also ensured that the osteotome inclination was maintained constant at 44°, determined from the rake angle of the osteotome cutting edge.

### Visual assessment

Qualitative visual assessment of osteotome cutting-edge condition entailed unmagnified visual inspection under bright natural light and included the acquisition of photographic images using a digital single lens reflex (DSLR) camera. Initial visual benchmarking was undertaken on all new osteotomes and then repeated after each of the four bone cutting cycles.

### Blade sharpness index

**Fig. 2:**
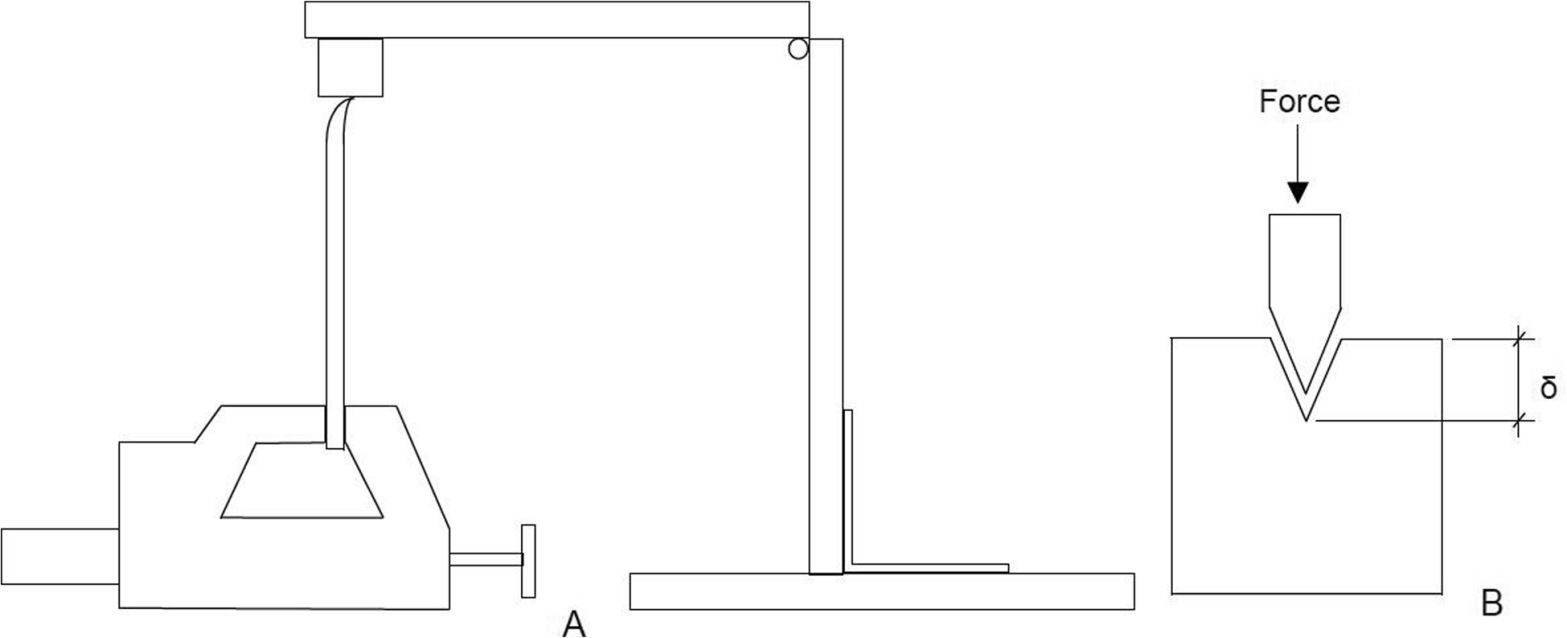
**a** Schematic of blade sharpness index testing using standardized force to press blade into soft wax substrate. Osteotome is shown clamped by vice in upward position with opposing soft wax substrate facing downward being located at end of hinged arm. **b** Blade penetration depth into soft wax substrate, measured by distance δ

The method adopted to qualitatively assess cutting-edge performance in this study was based on work by McCarthy et al. that applies the dimensionless BSI index number [3]. This procedure assesses blade sharpness using a depth penetration test where the osteotome cutting edge is forced into a soft wax substrate enabling objective evaluation of sharpness without damaging the fine edge. BSI can be regarded as a function of blade tip radius and force required to initiate the cut [4]. The test apparatus consisted of a hinged arm that allowed slight vertical motion with negligible change in angular position holding the osteotome with the cutting edge facing down into the wax substrate shown in Fig. 2a. A standardized load applied on top of the osteotome forced the osteotome cutting edge to penetrate the wax shown in Fig. 2b. The absolute value of the standardized load was of no significance as it was selected to cause sufficient blade penetration into the soft wax to enable comparison between sharp and blunt cutting-edge depth of penetration.

Initial sharpness benchmarking was undertaken on all new osteotomes. Sharpness assessments were then repeated after each of the four bone cutting cycles for all three osteotomes. Each wax sample was subsequently sectioned, and depth of blade penetration measured using an optical microscope, enabling the BSI to then be calculated [3].

### Scanning electron microscopy

Upon completion of all four bone cutting cycles and subsequent visual and BSI assessments, each osteotome was then thoroughly cleaned and sterilized before a small 5 mm × 10 mm sample of the cutting edge was removed by wire spark-erosion. Each cutting-edge sample then underwent SEM analysis (Hitachi SU-70) to qualitatively assess and compare the degree of wear and mechanical damage incurred to each osteotome blade upon completion of four bone cutting cycles.

## Results

### Visual assessment

**Fig. 3:**
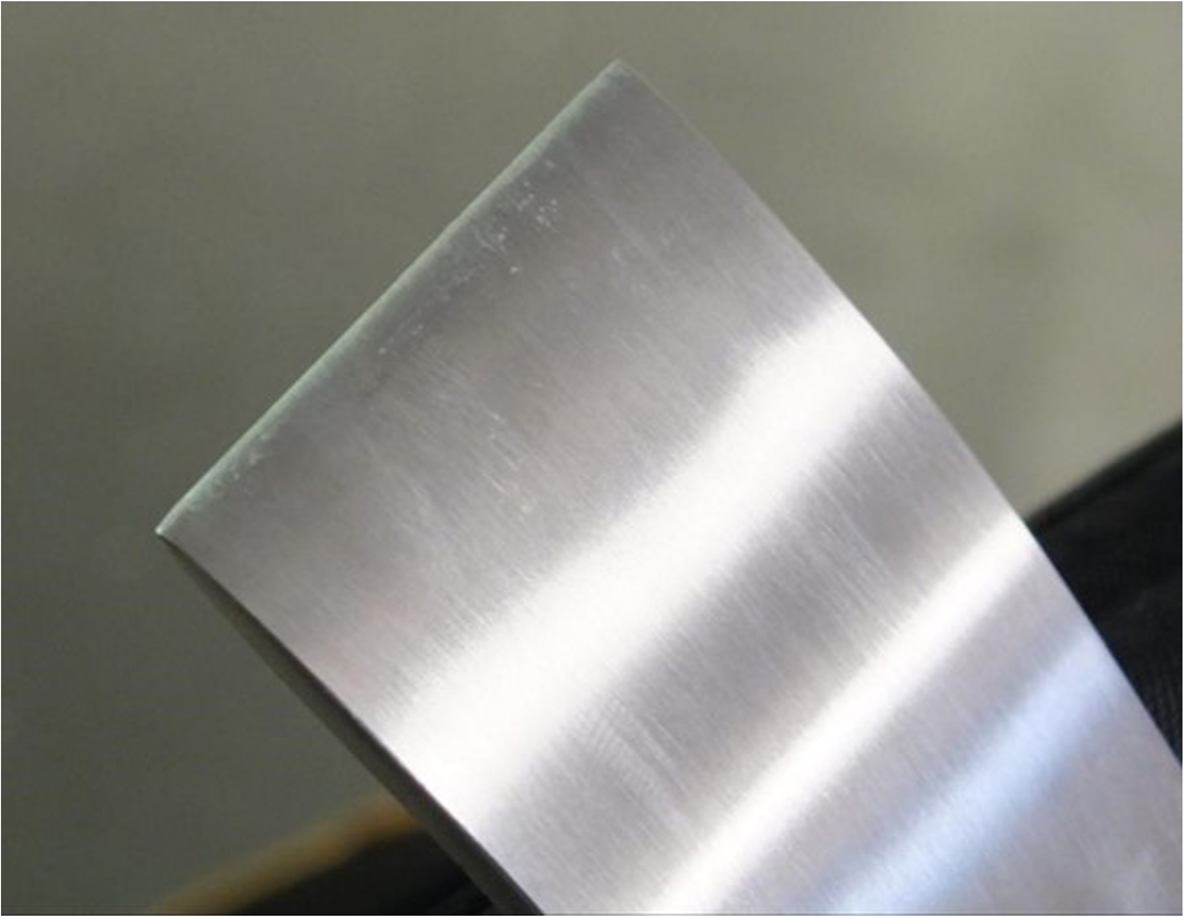
Image of as-supplied osteotome cutting edge upon completion of four bone cutting cycles displaying no evidence of loss of blade sharpness

Upon completion of all bone cutting tests, and after cleaning and sterilization, visual assessment failed to detect any degradation in cutting-edge condition in any of the osteotomes. Figure 3 presents an image of the as-supplied osteotome upon completion of four bone cutting cycles. There was no detectable difference in blade condition between any of the three osteotomes.

### Blade sharpness index

**Fig. 4:**
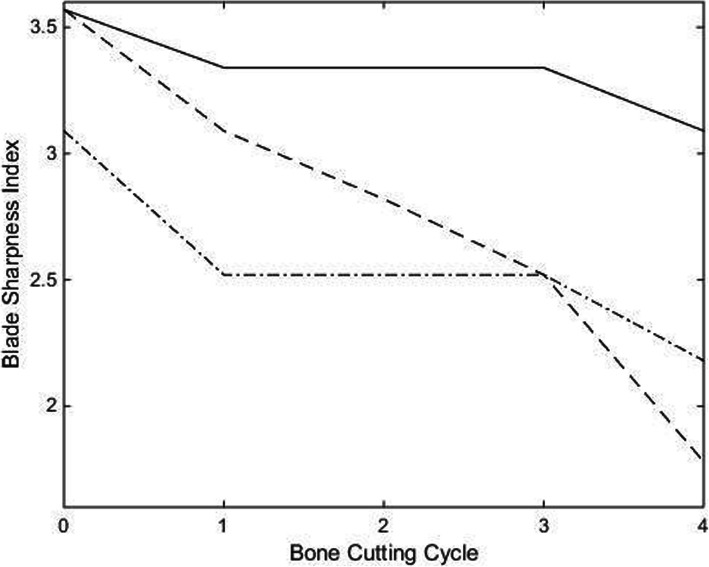
Trend of change in blade sharpness index from baseline spanning four bone cutting cycles. “” = TiN PVD coated osteotome, “” = electroless nickel coated osteotome, and “” = as-supplied osteotome

The trend in reduction of BSI, representing degradation in cutting edge between the as-supplied and coated osteotomes, is presented in Fig. 4. All osteotomes experienced loss of blade sharpness, with the TiN PVD coating demonstrating the best wear resistance. Of note is the lower baseline sharpness of the electroless nickel coated osteotome prior to undertaking bone cutting when compared to the other two.

**Fig. 5:**
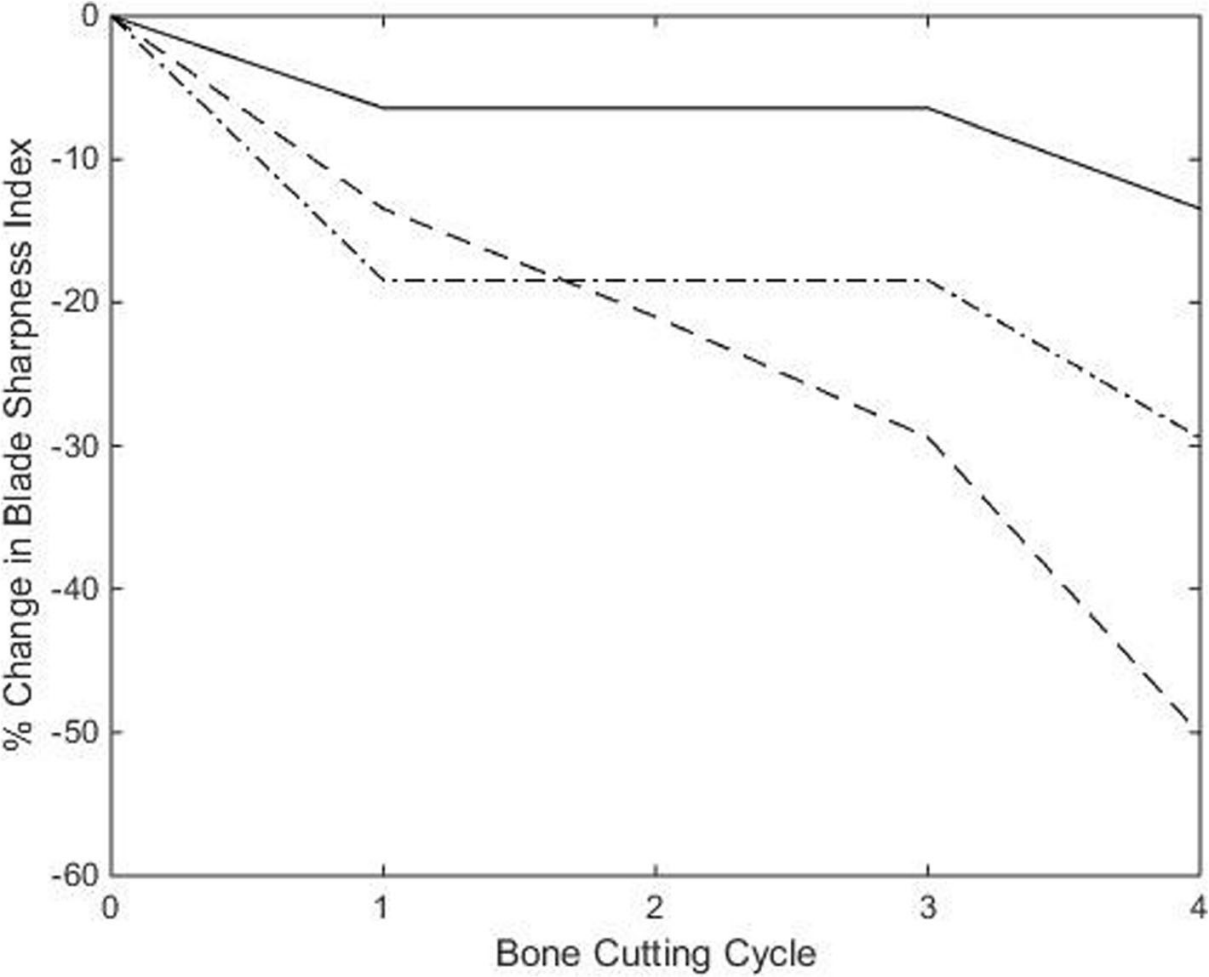
Percentage change from baseline blade sharpness index over four consecutive bone cutting cycles. “” = TiN PVD coated osteotome, “” = electroless nickel coated osteotome, and “” = as-supplied osteotome

Percentage change in BSI, presented in Fig. 5, demonstrates the rapid decline in blade sharpness experienced by the as-supplied osteotome compared to the two coated samples.

### Scanning electron microscopy examination

**Fig. 6:**
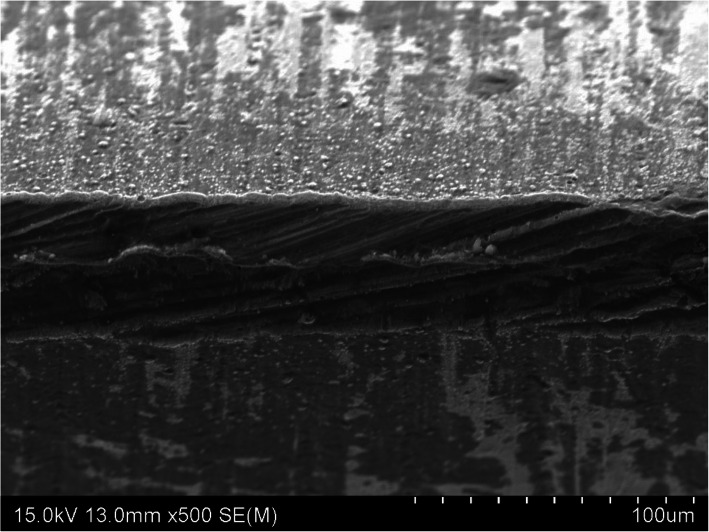
SEM image of the TiN PVD coated osteotome cutting edge shown in the middle of the image at × 500 magnification showing minor mechanical damage along the cutting edge and slight scratching along the face of blade

**Fig. 7:**
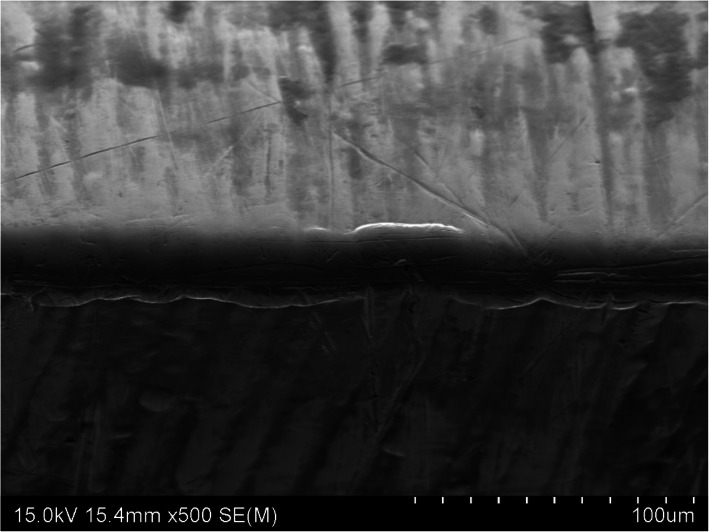
SEM image of the electroless nickel coated osteotome cutting edge shown in the middle of the image at × 500 magnification displaying rounded cutting edge and minor scratching along the face of blade

**Fig. 8:**
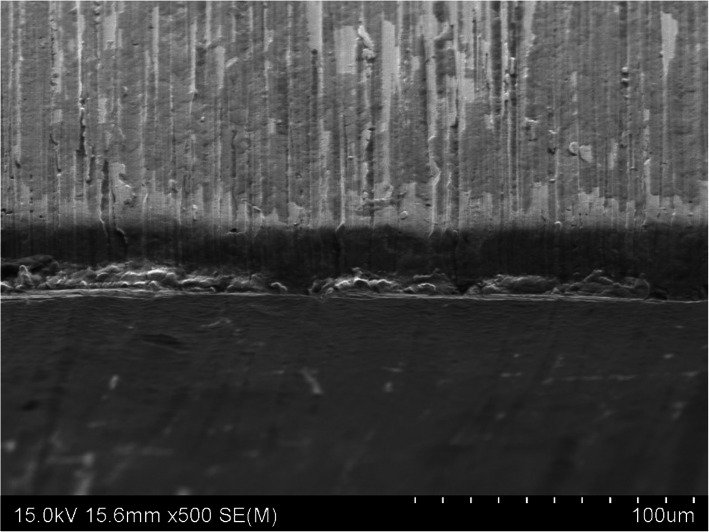
SEM image of the as-supplied osteotome cutting edge shown in the middle of the image at × 500 magnification showing significant mechanical damage along the cutting edge and deep scratching along the face of blade

SEM image × 500 magnification of the three osteotome blade samples identified different cutting-edge damage and variation in wear along the cutting face. The TiN PVD coated osteotome demonstrated a sharp cutting edge with minor mechanical damage and slight scratching along the face of blade shown in Fig. 6. This contrasted with the electroless nickel coated osteotome cutting edge that displayed a smooth rounded cutting edge and minor scratching along the blade face shown in Fig. 7. Of note is the as-supplied osteotome cutting edge that demonstrated both significant mechanical damage along the cutting edge and deep scratching along the blade face shown in Fig. 8.

## Discussion

### Visual inspection

Our study has shown that visual examination is unable to detect any degradation in the cutting edge of any of the three osteotomes tested. This includes the as-supplied osteotome that experienced a 50% loss in BSI over the four bone cutting tests. While visual inspection might detect catastrophic damage in the cutting edge, our study demonstrates this technique is unable to detect and gauge normal wear up to a level of 50% loss in BSI, as demonstrated in the as-supplied osteotome.

### Blade sharpness

There are currently various methods of maintaining osteotome sharpness. These range from periodic professional sharpening through to a surgeon hand sharpening the tool using ceramic or diamond sharpening stones or fine grit whetstones either prior to or during each use. Evaluation of the effectiveness of professionally sharpening blunt osteotomes has shown that edge sharpness is only ever at best partially restored [5].

Only two previous studies have investigated degradation in uncoated osteotome cutting performance and sharpness [1, 5]. Bloom et al. evaluated osteotome sharpness after repeated use, regular maintenance, and sharpening, by analyzing three identical osteotomes used in nasal dorsal hump reduction surgery [5]. In this study and after each use, degradation in osteotome blade sharpness was measured by holding the osteotome stationary while a single suture was incremented toward the blade with increasing force until the suture was broken. During this investigation, each osteotome was sharpened after three, six, and nine surgical uses. This entailed the first two osteotomes being hand sharpened by the surgeons using different methods while the third osteotome was professionally sharpened. The study found the cutting edge of all three osteotomes experienced significant deterioration in sharpness during use and that both hand and professional sharpening was unable to restore the cutting edge to its original degree of sharpness. Two hospital maintained osteotomes were included to compare the earlier findings with real-world conditions. Of note, the cutting edge of the hospital maintained osteotomes were significantly duller compared to the three osteotomes that had been tested 9 times. This study concluded that osteotomes may not be suitable for reuse, even after sharpening, and that edge retention is difficult to retain with repeated use [5]. A later study by the same group (Ransom et al.) focused on comparing the sharpness of osteotomes from five major manufacturers [1]. Like our study, this earlier investigation involved each osteotome making 40 mm cuts but using artificial rather than real bone. Baseline sharpness values were similarly determined for each new osteotome prior to change in sharpness being assessed after successive cutting cycles. Like our study for the as-supplied osteotome, each osteotome demonstrated progressive loss of cutting-edge sharpness, and degradation of the osteotome cutting edge was rapid and unavoidable.

### Hard coating

To improve osteotome cutting-edge durability, efforts have been focused on improving the wear resistance by applying a hard-wearing coating such as TiN before comparing their performance to uncoated as-supplied tools. Several authors have previously demonstrated the possible advantages of using coated instruments [6–9] but have so far neglected the osteotome.

In our study, baseline BSI values show the electroless nickel coating process leads to slight dulling of the cutting edge to a level equivalent to the as-supplied osteotome after one cutting cycle of use. This finding is supported by the post bone cutting SEM analysis that showed a smooth and rounded cutting edge shown in Fig. 7. This increase in cutting-edge radius is thought to be caused by the electroless nickel coating process. With the TiN PVD coating being much thinner, there was a lower increase in blade cutting-edge radius, leading to no difference in BSI between the TiN PVD coated and as-supplied osteotomes prior to use.

After four bone cutting cycles, the percentage change in BSI shows the as-supplied osteotome experiences the greatest cutting-edge wear rate compared to the other two osteotomes coated in wear resistant materials. This is demonstrated by the as-supplied osteotome having lost 50% of its BSI, which equates to requiring twice as much impact force to realize the same cut as when new. This compares to 10% and 35% loss of BSI for the TiN PVD and electroless nickel coated osteotomes, respectively. These findings are also supported by SEM examination of the cutting edges of each of the three osteotomes tested, with the TiN PVD coated osteotome showing the least cutting-edge mechanical damage and cutting face scratching.

## Limitation

This pilot investigation was limited to one sample of each osteotome, so any variation in performance is not detected. A prospective study investigating cutting blade edge degradation using a greater sample size should be conducted.

## Conclusions

Visual inspection does not detect osteotome cutting-edge wear up to a level of 50% loss in BSI. Other means of in-field assessment of blade sharpness should be considered to better assess blade sharpness prior to osteotome reuse. This could include BSI testing after each surgical procedure to monitor the inevitable loss in sharpness.

TiN PVD coating provides superior cutting-edge wear and damage resistance compared to electroless nickel coated and as-supplied osteotomes and should be considered for use by manufacturers as a cost-effective way to improve osteotome blade durability and reduce the need for sharpness testing after each use. Further study using a larger sample size is required to validate these findings.

## Data Availability

The data sets used and/or analyzed during the study are available from the corresponding author upon reasonable request.

## Abbreviations

BSI: Blade sharpness index
DSLR: Digital single lens reflex
PVD: Physical vapor deposition
SEM: Scanning electron microscopy
TiN: Titanium nitride
